# Transcriptome analysis of a barley breeding program examines gene expression diversity and reveals target genes for malting quality improvement

**DOI:** 10.1186/1471-2164-11-653

**Published:** 2010-11-23

**Authors:** María Muñoz-Amatriaín, Yanwen Xiong, Mark R Schmitt, Hatice Bilgic, Allen D Budde, Shiaoman Chao, Kevin P Smith, Gary J Muehlbauer

**Affiliations:** 1 Department of Agronomy and Plant Genetics, University of Minnesota, St. Paul, MN 55108, USA; 2 Pioneer Hi-Bred Int'l Inc., 810 Sugar Grove Ave, Dallas Center, IA 50063, USA; 3 Cereal Crop Research Unit, USDA-ARS, 502 Walnut St., Madison, WI 53726, USA; 4 Department of Medicine/Rheumatology, University of Minnesota, Minneapolis, MN 55455, USA; 5 USDA-ARS Biosciences Research Lab, Fargo, ND, 58105-5674, USA

## Abstract

**Background:**

Advanced cycle breeding utilizes crosses among elite lines and is a successful method to develop new inbreds. However, it results in a reduction in genetic diversity within the breeding population. The development of malting barley varieties requires the adherence to a narrow malting quality profile and thus the use of advanced cycle breeding strategies. Although attention has been focused on diversity in gene expression and its association with genetic diversity, there are no studies performed in a single breeding program examining the implications that consecutive cycles of breeding have on gene expression variation and identifying the variability still available for future improvement.

**Results:**

Fifteen lines representing the historically important six-rowed malting barley breeding program of the University of Minnesota were genotyped with 1,524 SNPs, phenotypically examined for six malting quality traits, and analyzed for transcript accumulation during germination using the Barley1 GeneChip array. Significant correlation was detected between genetic and transcript-level variation. We observed a reduction in both genetic and gene expression diversity through the breeding process, although the expression of many genes have not been fixed. A high number of quality-related genes whose expression was fixed during the breeding process was identified, indicating that much of the diversity reduction was associated with the improvement of the complex phenotype "malting quality", the main goal of the University of Minnesota breeding program. We also identified 49 differentially expressed genes between the most recent lines of the program that were correlated with one or more of the six primary malting quality traits. These genes constitute potential targets for the improvement of malting quality within the breeding program.

**Conclusions:**

The present study shows the repercussion of advanced cycle breeding on gene expression diversity within an important barley breeding program. A reduction in gene expression diversity was detected, although there is diversity still present after forty years of breeding that can exploited for future crop improvement. In addition, the identification of candidate genes for enhancing malting quality may be used to optimize the selection of targets for further improvements in this economically important phenotype.

## Background

Genetic diversity within breeding populations is indispensable for obtaining genetic gains, and consequently for plant breeding progress. Plant breeding that involves crossing elite lines in a closed population is called advanced cycle breeding [1] and it has proved to be successful in achieving genetic gains in major crops such as barley (*Hordeum vulgare *L.), maize (*Zea mays *L.), rice (*Oryza sativa *L.), soybean (*Glycine max *L. Merr.) and wheat (*Triticum aestivum *L.) ([2] and references therein). However, over cycles of selection, the genetic variability within breeding populations is reduced, presumably reducing the potential for future gains and increasing genetic vulnerability [3-7]. Therefore, an evaluation of an ongoing breeding program is necessary to gain an understanding of the existing diversity and optimize current and future improvements. At present, few studies have evaluated the variation in genetic diversity within a single breeding program, with examples including barley [3] and wheat [8].

The development of new barley varieties with improved malting quality characteristics is one of the primary aims of the US barley breeding programs due to the economic impact of the malting and brewing sector. Malting quality is a genetically complex phenotype representing a set of component traits, many of which are interrelated [9-11]. Grain protein content, malt extract percentage, ratio of wort soluble protein to total malt protein, diastatic power, α-amylase activity, and wort β-glucan content are some of the most important parameters contributing to malting quality. Quality-related QTL reported in the literature have been recently summarized, resulting in 154 QTL associated with 18 quality traits that are located on all barley chromosomes [12], which reveals the genetic complexity of this phenotype. In the U.S., the malting and brewing industry require that new cultivars meet quality parameters specified by maltsters and brewers that generally agree with the ideal commercial malt criteria established by the American Malting Barley Association (AMBA) http://www.ambainc.org/media/AMBA_PDFs/Press_Releases/GUIDELINES.pdf. This, together with the complexity of the phenotype and the high cost of malting quality evaluation, has encouraged breeders to follow a conservative strategy, favoring crosses among closely related elite cultivars with good quality characteristics in order to maintain acceptable malting performance [13,14].

The University of Minnesota six-rowed malting breeding program has used advanced cycle breeding since 1958, developing important malting cultivars like the variety "Morex", an archetype for malting quality, and the AMBA recommended six-rowed malting cultivars "Robust", "Lacey" and the recently released "Rasmusson" http://www.ambainc.org/media/AMBA_PDFs/Pubs/KYMBV_2010.pdf. This closed population offers an excellent opportunity to study the effect of plant breeding on genetic gain, genetic diversity and phenotypic variation. Condón et al. [3] examined the effect of advanced cycle breeding on allelic diversity and showed a reduction in the number of alleles per locus, from an average of 5.89 to 2.34. This reduction was not uniform across the genome, predictably due to selection pressure on disease-resistance and quality traits. However, only 28% of the total loci studied had been fixed, indicating that there is still genetic variability in the University of Minnesota elite germplasm that can be exploited. Genetic gains during advanced cycle breeding were documented by the same group for most of the 15 agronomic and malting quality traits evaluated [13]. Of the seven traits whose phenotypic variance changed over the four decades of advanced cycle breeding, five showed a significant decrease. Both studies reported that the breeding process generated a germplasm differentiation between the most recent genotypes and their ancestors.

Recently, increased attention has been paid to the influence of gene expression differences on phenotypic variation. In Arabidopsis, the Affymetrix ATH1 GeneChip was used to analyze the gene expression diversity between seven pairs of accessions [15]. This study showed that 10-30% of the Arabidopsis genome exhibited natural transcript-level variation. In addition, there was a positive correlation between DNA sequence polymorphisms and gene expression differences. Although this is probably the most exhaustive study of gene expression diversity, other groups have also mentioned differences in gene expression in five maize inbred lines [16] and four barley varieties [17]. The percentage of the transcriptome showing variation in gene expression was much smaller in these two cases, approximately 1.6-6% in maize and 0.1-1.5% in barley. Stupar et al. [16] also found a strong correlation between genetic diversity and differential gene expression data. However, all these studies used unrelated collections of genotypes and the gene expression diversity between lines in a breeding program is unknown.

Approaches using transcriptome analysis have been used to examine gene expression during the early stages of germination [18-21] and to find candidate genes for malting quality [17,22]. Potokina et al. [22] used ten well-characterized barley cultivars to correlate gene expression patterns with six malting-quality parameters, and identified 49 genes that included both well-known and unknown malting-related genes. More recently, Lapitan et al. [17] used the Barley1 GeneChip in a set of four North American malting varieties with the same purpose, and identified genes correlated with six malting quality phenotypes. However, there have been no gene expression studies performed on members of a malting barley breeding program. Such studies would characterize variation in gene expression diversity during advanced cycle breeding, and also identify the variability still available for future progress on malting quality traits within a given breeding program.

In this study, we used the Barley1 GeneChip to analyze the transcriptome patterns during germination of fifteen lines representing the historically important six-rowed malting barley breeding program of the University of Minnesota. The objectives were to: (1) examine the effect of advanced cycle breeding on gene expression diversity; (2) identify the quality-related genes whose expression has been fixed during the breeding process; and (3) identify genes that represent potential targets for improving malting quality.

## Methods

### Plant material and micromalting conditions

**Figure 1:**
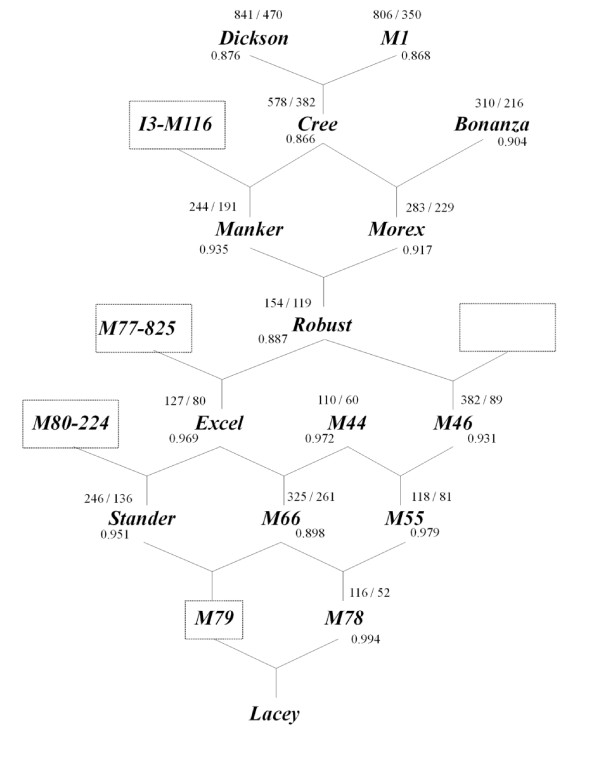
**Pedigree information for the University of Minnesota barley genotypes used in this study**. The number of differentially expressed genes at "out of steep" and "3d of germination" relating to Lacey is placed above each line, while the number below each genotype indicates the Genetic similarity (GS) index related to Lacey. Boxes represent genotypes not included in the study. The empty box refers to an unknown genotype.

For this study, fifteen six-rowed malting barley genotypes were used. These genotypes represent the University of Minnesota core germplasm, and include: the parental lines (PL) Dickson and Bonanza; the elite lines developed between 1958 and 1967 (EL58-67) M1, Cree and Manker; the EL68-77 Morex, Robust and M44; the EL78-87 M46, Excel, M55, Stander and M66; and the EL88-98 M78 and Lacey, as described by [3]. Figure 1 shows the pedigree information of the 15 genotypes, which are a subset of the entire Minnesota breeding program whose pedigree can be found in [3]. Breeding lines were advanced by single-seed-decent to the F5 generation. Selection began in the F4 generation for plant height, maturity, lodging resistance, and straw strength. This was followed by 3-4 years of replicated field trials selecting for yield and malting quality.

The fifteen genotypes were grown in a randomized complete block experiment with three replicates in single row plots 2 m in length in St Paul, MN in the summer of 2005. The plots were harvested at maturity with a small plot combine (Wintesteiger, Ried, Germany) and the seed cleaned using a Sample Cleaner model SLN4 (A/S Rational Korn Service, Esbjerg, Denmark). The cleaned seed from the three replicates was bulked and mixed thoroughly for micromalting. Micromalting was performed at the USDA-ARS Barley and Malt Laboratory of the Cereal Crops Research Unit (CCRU) in Madison, Wisconsin, under standard CCRU micromalting conditions. Barley grains (175 grams) were steeped at 16°C with individual steep times adjusted from 24 to 32 hours to achieve a homogeneous steep-out moisture of 45%. This uniform grain moisture is necessary to equalize the starting point of the malting process and, hence, establish a comparable starting physiological state across the lines. Steeping involved alternating 4-hour periods of immersion in water and air-rest. The hydrated grains were transferred to germinators immediately after steeping and maintained at 17°C and 100% relative humidity in the dark. Samples were collected at the end of the steep period ("out of steep") and after three days in the germinator ("3d of germination"). Three samples for each time point and genotype were harvested and frozen in liquid nitrogen, and cleaned of emergent rootlets and acrospires prior to microarray analysis. Finished malts derived from this experiment were subjected to malting quality analysis. A total of six parameters commonly used to assess malt quality were measured following the standard ASBC (American Society of Brewing Chemists 2004) procedures: malt extract percentage (ME), grain protein content (GP), ratio of soluble protein to total malt protein (S/T), diastatic power (DP), α-amylase activity (AA), and wort β-glucan concentration (BG). ME is one of the most important parameters for maltsters and brewers and measures the amount of soluble sugars and nitrogenous compounds obtained upon mashing malt into wort [9], which is directly related to alcohol production during fermentation and hence brewery profitability. GP is a measure of the percentage of protein found in the grain at maturity (prior to malting) and influences many of the other quality traits. S/T reflects proteinase activity and the extent of protein mobilization. AA and DP are measures of the carbohydrate-degrading enzymatic activities in malt. DP includes the combined action of alpha-amylase, beta-amylase and the rest of the amylolytic enzymes [9]. Finally, BG reflects the amount of cell wall breakdown products present in wort after mashing, and provides a measure of the extent of malt modification.

To ensure that the malting profile of the samples used in this study was representative of the general performance of these lines, we examined malting quality data collected from field trials published in a previous study [13]. These prior malting quality data, based on the mean of five trials conducted in Minnesota in 2002 and 2003, are presented in Additional file 1.

### RNA isolation, labeling and GeneChip hybridization

Total RNA was isolated from the ninety grain samples (fifteen genotypes, two time points, and three replicates) using TRIzol^® ^Reagent (Invitrogen, Carlsbad, CA), with a phenol/chloroform/isoamyl alcohol (49:49:2) pre-treatment extraction step and passed through RNeasy columns (Qiagen, Valencia, CA) for further clean up. Prior to labeling, RNA quality was examined by means of an Agilent Bioanalyzer 2100 (Agilent Technologies, Palo Alto, CA). cDNA synthesis was conducted with 15 μg of total RNA and T7-Oligo(dT) primer (Proligo, Boulder, CO) using the SuperScript Double-Stranded cDNA Synthesis Kit (Invitrogen, Carlsbad, CA). The cDNA was purified with the Affymetrix GeneChip Sample Cleanup Module (Affymetrix, Santa Clara, CA). To produce biotinylated cRNA, the cDNA was transcribed *in vitro *using the Enzo BioArray HighYield RNA Transcript Labeling Kit (Enzo Life Sciences, Farmingdale, NY) in the presence of biotinylated UTP and CTP. The biotin-labeled cRNA was purified with the Affymetrix GeneChip Sample Cleanup Module (Affymetrix, Santa Clara, CA). Labeled RNA (15 μg) was chemically fragmented using the Affymetrix GeneChip Sample Cleanup Module (Affymetrix), and used for hybridization. The chip hybridizations, washes, and data acquisition were conducted at the Biomedical Image Processing Facility at the University of Minnesota following standard Affymetrix procedures. Data files are available online at PLEXdb (experiment BB91, http://www.plexdb.org/modules/PD_browse/experiment_browser.php?experiment=BB91) and at NCBI-GEO (accession number GSE24534, http://www.ncbi.nlm.nih.gov/geo/query/acc.cgi?acc=GSE24534).

### Data analysis

GeneChip data were analyzed using the software GeneSpring GX 9.0 (Silicon Genetics, Redwood City, CA, USA). Expression estimates were calculated using the gcRMA algorithm implemented in GeneSpring. Quality control on samples was performed using various criteria including internal control 3'/5' ratio, sample correlation matrix, principal component analysis (PCA), and hierarchical clustering. One replicate of genotype M44 and another replicate of genotype M55 were removed for subsequent analyses based on their correlation coefficients and position in the PCA and hierarchical clustering. Only probe sets with a signal intensity value greater than the 20^th ^percentile in all replicates for at least one condition were considered reliable and included for further analysis. Differentially expressed genes between the fifteen genotypes were identified using one-way ANOVA with Benjamini-Hochberg false discovery rate (FDR) correction [23]. Analysis was done using a FDR adjusted *P *value of 0.01 as the cutoff, followed by filtering for two-fold or greater changes. A SNK post-hoc test was applied to identify the genes differentially expressed by pairs of genotypes. For annotation purposes, BLASTX (E-score cutoff = e^-10^) data was exported from HarvEST:Barley version 1.68 http://harvest.ucr.edu/. To associate gene expression with malting quality phenotypic data, Pearson's correlation coefficients were calculated for each of the six malting quality parameters using the normalized intensity values averaged over replicates. Analysis was carried out with the use of SAS software, version 9.1 (SAS Institute Inc., Cary, NC, USA).

The fifteen lines were genotyped with 1,524 SNPs from the pilot Oligonucleotide Pool Assay 1 (POPA1) [24] using the Illumina GoldenGate assay. Genotyping was conducted at the USDA-ARS small grains genotyping center at Fargo, North Dakota. Genetic similarities between the fifteen genotypes were calculated using the genetic similarity (GS) index proposed by Nei and Li [25] with POPA1 SNPs. Correlation coefficients between genetic similarities and the number of differentially expressed genes between genotypes were also calculated using Pearson's correlation implemented in SAS software (SAS Institute Inc., Cary, NC, USA).

### Single-feature polymorphism (SFP) analysis

Oligonucleotide arrays such as the Barley1 GeneChip can be used to detect single-feature polymorphisms or SFPs [26]. To test if SFPs significantly affected gene expression estimates and, therefore, the detection of gene expression differences between genotypes, we compared the list of differentially expressed genes between Dickson and Bonanza at time point "out of steep" before and after removing the probes that contained SFPs. We chose these two genotypes as they showed the largest number of differentially expressed genes. SFPs were detected using the probe affinity shape power method described by Xu et al. [27]. After masking the SFP probes, differentially expressed genes between the two genotypes were identified by *t*-test with a FDR adjusted p-value of 0.01, followed by filtering for two-fold or greater changes. A venn diagram was used to compare the probe sets contained in both lists, showing that 96.3% of the genes were common. This revealed that SFPs do not significantly alter the perceived expression differences as had already been described in Arabidopsis by Kliebenstein et al. [15]. Therefore, probes with SFPs were not excluded from the analysis.

## Results

### Genetic, phenotypic and functional characterization of a barley breeding program

We phenotypically, genetically and functionally characterized 15 lines representing the University of Minnesota malting barley breeding program (Figure 1). Since the development of new improved malting varieties is the main goal of the breeding program, we analyzed the transcriptome of key members of the program at two key stages of the malting process, "out of steep" and "3d of germination", by means of the Barley1 GeneChip [28]. Using a FDR adjusted *P *value of 0.01 and filtering by two-fold or greater changes, statistical analysis resulted in a total of 1,442 differentially expressed genes between the 15 lines at time point "out of steep", and 851 genes at "3d of germination". Post-hoc comparisons using the SNK test showed that Dickson and Bonanza were the most diverse lines in terms of gene expression since they had the highest number of differentially expressed genes at both "out of steep" and "3d of germination" (1,005 and 558 genes, respectively; Tables 1 and 2). On the contrary, the most similar lines at "out of steep" were Robust and M78, as they showed the smallest number of differentially expressed genes (57) (Table 1), while M44 and M46 were the most similar lines at "3d of germination", with only 3 differentially expressed genes (Table 2). All pair-wise comparisons between genotypes are shown in Table 1, for time point "out of steep", and Table 2, for "3d of germination".

**Table 1:** Number of differentially expressed genes between the fifteen genotypes at time point "out of steep" in pair-wise comparisons.

	Bonanza	Cree	Dickson	Excel	Lacey	M1	M44	M46	M55	M66	M78	Manker	Morex	Robust	Stander
**Bonanza**	0														
**Cree**	691	0													
**Dickson**	1005	353	0												
**Excel**	337	524	771	0											
**Lacey**	310	578	841	127	0										
**M1**	902	177	283	692	806	0									
**M44**	351	604	853	182	110	827	0								
**M46**	579	357	439	288	382	400	409	0							
**M55**	385	485	718	93	118	647	131	255	0						
**M66**	405	661	923	359	325	866	336	610	397	0					
**M78**	386	445	642	81	116	579	172	226	100	398	0				
**Manker**	491	545	718	226	244	699	169	405	198	413	234	0			
**Morex**	295	521	865	338	283	799	360	495	360	374	306	496	0		
**Robust**	350	424	691	112	154	625	218	220	125	422	57	264	270	0	
**Stander**	457	474	611	106	246	596	252	221	158	432	128	252	380	167	0

These numbers were determined based on ANOVA (FDR ≤ 0.01) followed by a two-fold filtering and a SNK post-hoc test.

**Table 2:** Number of differentially expressed genes between the fifteen genotypes at time point "3d of germination" in pair-wise comparisons.

	Bonanza	Cree	Dickson	Excel	Lacey	M1	M44	M46	M55	M66	M78	Manker	Morex	Robust	Stander
**Bonanza**	0														
**Cree**	431	0													
**Dickson**	558	288	0												
**Excel**	208	368	463	0											
**Lacey**	216	382	470	80	0										
**M1**	388	96	346	339	350	0									
**M44**	210	289	382	72	60	272	0								
**M46**	218	276	362	88	89	268	3	0							
**M55**	256	387	475	68	81	352	65	81	0						
**M66**	259	426	537	238	261	413	238	259	277	0					
**M78**	204	337	450	58	52	304	47	66	75	255	0				
**Manker**	337	372	450	177	191	347	142	171	158	310	181	0			
**Morex**	226	269	413	235	229	266	178	183	261	290	166	371	0		
**Robust**	245	330	439	87	119	302	79	92	101	290	46	193	194	0	
**Stander**	249	358	440	69	136	335	85	97	103	263	99	180	221	121	0

These numbers were determined based on ANOVA (FDR ≤ 0.01) followed by a two-fold filtering and a SNK post-hoc test.

The genetic characterization of the 15 lines was done using 1,524 SNPs available from POPA1 [24]. Pair-wise genetic similarity indexes were calculated between the fifteen lines and values ranged from 0.829 to 0.994 (Table 3). The lowest similarity coefficient was observed between Dickson and Bonanza, indicating that these lines were not only the most different at the level of gene expression but also the most genetically different. The genetically closest lines were M78 and Lacey. These lines occur late in the breeding program history and M78 is one of the parents of Lacey (Figure 1).

**Table 3:** Genetic similarity (GS) indexes between the fifteen genotypes.

	Bonanza	Cree	Dickson	Excel	Lacey	M1	M44	M46	M55	M66	M78	Manker	Morex	Robust	Stander
**Bonanza**	1														
**Cree**	0.845	1													
**Dickson**	0.829	0.954	1												
**Excel**	0.906	0.871	0.879	1											
**Lacey**	0.904	0.866	0.876	0.969	1										
**M1**	0.853	0.988	0.944	0.872	0.868	1									
**M44**	0.903	0.864	0.882	0.943	0.972	0.866	1								
**M46**	0.888	0.901	0.910	0.934	0.931	0.895	0.922	1							
**M55**	0.886	0.875	0.888	0.972	0.979	0.876	0.960	0.937	1						
**M66**	0.872	0.855	0.861	0.900	0.898	0.851	0.900	0.862	0.884	1					
**M78**	0.903	0.865	0.875	0.964	0.994	0.865	0.973	0.930	0.980	0.897	1				
**Manker**	0.863	0.893	0.915	0.931	0.935	0.900	0.947	0.917	0.946	0.878	0.932	1			
**Morex**	0.911	0.903	0.895	0.907	0.917	0.896	0.901	0.910	0.906	0.889	0.918	0.866	1		
**Robust**	0.900	0.884	0.870	0.874	0.887	0.887	0.886	0.891	0.880	0.884	0.888	0.859	0.914	1	
**Stander**	0.910	0.884	0.894	0.967	0.951	0.877	0.934	0.939	0.956	0.899	0.945	0.927	0.920	0.887	1

These indexed were calculated based on 1,524-POPA1 SNPs.

To examine the malting quality characteristics of the lines under study, a total of six quality-assurance parameters were considered. Values for the measured parameters were obtained from finished malts derived from grain used in the microarray experiments and are shown in Table 4. Mean values corresponding to five field trials accomplished in three different locations of Minnesota during 2002-03 [13] are also presented to provide a general view of the quality performance of the genotypes (Additional file 1). In general, the data from the microarray experiment were consistent with the previous study. Correlations between the two data sets were 0.86, 0.30, 0.82, 0.50, 0.92, and 0.77 for ME, GP, S/T, DP, AA, and BG, respectively.

**Table 4:** Malting quality characteristics of the fifteen lines used in this study.

	ME (%)	GP (%)	S/T (%)	DP (°ASBC)	AA (20°DU)	BG (ppm)
Dickson	74.5 ± 0.2	14.6 ± 0.0	34.7 ± 1.0	144.5 ± 3.5	48.4 ± 1.5	431.3 ± 13.5
M1	73.8 ± 0.3	14.2 ± 0.2	35.5 ± 0.1	141.0 ± 6.7	50.9 ± 2.5	377.3 ± 12.5
Cree	74.5 ± 0.3	14.5 ± 0.0	36.2 ± 1.8	163.9 ± 4.1	51.3 ± 1.1	395.7 ± 5.6
Bonanza	76.1 ± 0.3	15.8 ± 0.0	37.1 ± 0.5	160.2 ± 3.9	64.2 ± 2.5	197.9 ± 16.7
Manker	75.6 ± 0.3	14.6 ± 0.0	44.8 ± 0.7	130.0 ± 2.7	61.4 ± 3.0	401.3 ± 36.3
Morex	77.6 ± 0.3	14.2 ± 0.0	40.3 ± 0.0	173.0 ± 4.8	71.5 ± 2.0	199.4 ± 20.4
M46	76.4 ± 0.4	15.8 ± 0.0	40.2 ± 0.7	202.0 ± 6.2	63.4 ± 1.9	278.4 ± 14.4
M66	77.1 ± 0.3	14.9 ± 0.0	41.1 ± 0.8	162.1 ± 12.5	72.8 ± 1.1	357.2 ± 19.3
Robust	76.8 ± 0.2	14.7 ± 0.0	40.9 ± 0.7	169.9 ± 15.3	57.2 ± 2.5	291.3 ± 21.9
M44	75.4 ± 0.3	17.2 ± 0.0	39.8 ± 1.6	210.0 ± 7.8	67.2 ± 3.9	253.3 ± 10.4
Excel	77.4 ± 0.2	14.9 ± 0.2	44.1 ± 0.6	143.9 ± 19.9	67.5 ± 1.8	370.1 ± 4.2
M55	77.3 ± 0.4	15.1 ± 0.1	41.6 ± 0.5	167.8 ± 10.1	68.8 ± 1.4	211.3 ± 14.9
Stander	77.4 ± 0.4	15.3 ± 0.0	45.1 ± 0.7	166.8 ± 16.5	78.1 ± 1.9	267.1 ± 1.4
M78	77.2 ± 0.3	15.3 ± 0.2	39.4 ± 0.3	169.7 ± 12.9	64.2 ± 1.2	283.1 ± 20.4
Lacey	76.9 ± 0.2	15.6 ± 0.0	38.6 ± 0.9	191.7 ± 21.6	66.9 ± 0.8	164.4 ± 16.6

Data derives from the grain used for the microarray experiments. Means (three biological replicates) and standard deviations for each of the six malting quality traits are shown. ME: malt extract, GP: grain protein content, S/T: ratio of wort soluble protein to total malt protein, DP: diastatic power, AA: alpha-amylase activity, BG: malt beta-glucan content.

### Effect of advanced cycle breeding on gene expression diversity

**Figure 2:**
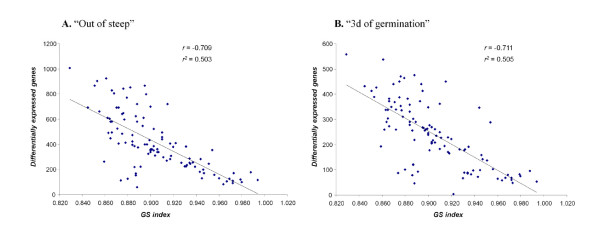
**Linear regression graphs of correlation between genetic similarities (GS) and number of differentially expressed genes**. Correlation coefficient (*r*) and coefficient of determination (*r*^*2*^) are shown for time point "Out of steep" (A) and "3d of germination" (B).

To examine the relationship between genetic and the gene expression data we performed a linear regression analysis and calculated the correlation coefficient (*r*) for both time points (Figure 2). Significant correlations were detected between genetic similarities and the number of differentially expressed genes between lines at "out of steep" (*r *= -0.709; *p *< .0001) and at "3d of germination" (*r *= -0.711; *p *< .0001).

To study the effect that advanced cycle breeding exerts on gene expression diversity, we focused on the number of differentially expressed genes between each line and the last cultivar released by the breeding program (Lacey). The number of transcripts identified for each time point was added to the figure where the pedigrees are represented (Figure 1), together with the genetic similarity (GS) indexes relating to Lacey. Interestingly, in all cases, there were more transcripts differentially expressed at "out of steep" compared to "3d of germination".

In general, we observed that as the breeding program progressed, the genetic similarities became higher while the number of differentially expressed genes became smaller (Figure 1), indicating a reduction in both genetic and gene expression diversity through the breeding process. The only exceptions were: Robust, whose similarity coefficient with Lacey is lower than both of its parents; and M66, in which both genetic and gene expression data indicate that it is more different from Lacey than its parents. In the case of Robust, our similarity coefficient was not in accordance with the gene expression data. Interestingly, we found a higher number of differentially expressed genes between one of the parental lines (Dickson) and the last developed cultivar (Lacey) compared to the other parental line (Bonanza) and Lacey. The GS indexes in Bonanza and Dickson were in accordance with these gene expression values (Figure 1).

The expression of many genes has been fixed through the advanced cycle breeding as can be observed by the reduction in the number of differentially expressed genes in the most recently developed lines (Figure 1). However, there are still many genes whose expression has not been fixed yet, as demonstrated by the relatively high number of differentially expressed genes (116 genes at time point "out of steep" and 52 genes at "3d of germination") that were identified between the two most recently developed lines of the breeding program, M78 and Lacey.

### Transcript accumulation correlated with malting quality traits

To identify candidate genes for the improvement of malting quality in the University of Minnesota breeding program, we used the following strategy: we selected the most recent lines of the breeding program based on their similarities to Lacey and we identified the differentially expressed genes between them that were associated with malting quality. The choice of these "most recent" lines was done considering genetic, gene expression and phenotypic data. Based on our similarity coefficients and number of differentially expressed genes related to Lacey (Figure 1), this group of genotypes includes M78, M55, Stander, Excel and M44 together with Lacey. We also focused on the Condón et al. [3] study since it included the lines used for this work. Using SSR data, these authors conducted a principle correspondence analysis in which Robust was located together with the previously mentioned lines. While the genetic similarity between Robust and Lacey was not very high (0.887), the numbers of differentially expressed genes were relatively small. Phenotypic data also supported the choice of Robust due to its importance as a malting cultivar, prevailing as a recommended variety and dominating US malting barley acreage for over 20 years ([5]; http://www.ambainc.org/media/AMBA_PDFs/Pubs/KYMBV_2010.pdf). Using these criteria, the group of most recent lines was expanded to include Robust, M44, Excel, Stander, M55, M78 and Lacey. The genes that are differentially expressed between these genotypes was determined by a one-way ANOVA (FDR adjusted *P *value ≤0.01) followed by a ≥2-fold filtering, and resulted in the identification of 86 and 39 genes at "out of steep" and "3d of germination", respectively.

Correlation between expression values of these genes and the six parameters used to assess malting quality was examined. A total of 37 of the 86 genes were found to be associated with one or more of the malting quality traits at "out of steep", while 25 of the 39 genes were correlated to any of the six parameters at "3d of germination" (Table 5). Thirteen genes were common in both time points, therefore 49 candidate genes were identified for the improvement of malting quality. Almost all common genes correlated with the same malting quality parameters in both time points. Alpha-amylase activity (AA) and ME were the quality traits with the highest number of correlated genes (25 and 20 genes, respectively). Fourteen genes were associated with GP and with BG, and 13 genes were correlated to S/T. Diastatic power had the lowest number of associated genes (6). Half of these 49 total target genes had no match in any database or coded for proteins with unknown function, and the classified genes encoded proteins with very different functions (Table 5). However, among the transcripts with known function that are correlated with malting quality we identified a hordoindoline, together with three core histones H4 and five proteolytic enzymes, three of them with serine-peptidase activity (Table 5).

**Table 5:** Candidate genes for the improvement of malting quality.

Probe Set Name	*E *value	Putative function	ME	GP	S/T	DP	AA	BG	**Map loc**.
*"Out of steep"*									

Contig10584_at	_	No hit	**(+)						
**Contig12460_at**	7.00E-22	Hypothetical protein P0657H12.23 (*Oryza sativa*)		**(-)				*(+)	
Contig13150_at	_	No hit			*(+)				
**Contig14046_at**	1.00E-21	Hypothetical protein OJ1756_H07 (*Oryza sativa*)			*(-)		*(-)		
**Contig17218_at**	**_**	No hit	**(+)				*(+)		
**Contig17647_at**	2.00E-29	Os08g0100400 protein	*(-)				*(-)		
Contig21675_at	_	No hit	***(+)				**(+)	*(-)	
**Contig22323_at**	6.00E-38	26S protease regulatory subunit 6B	**(+)				*(+)		
Contig23156_at	_	No hit		*(-)		*(-)		**(+)	
Contig25703_at	2.00E-32	F-box domain containing protein	**(+)					*(-)	
**Contig2885_at**	4.00E-60	Eukaryotic translation initiation factor 5 (*Zea mays*)		*(+)				**(-)	
**Contig3129_at**	1.00E-124	Mitochondrial prohibitin complex protein 2 (*Oryza sativa*)	*(-)		**(-)		*(-)		
Contig3173_at	2.00E-23	Low temperature and salt responsive protein (*Pennisetum americanum*)						*(+)	2H (51.75)
Contig3191_at	1.00E-121	Mitochondrial ATP synthase (*Triticum aestivum*)					*(+)		
Contig3339_at	1.00E-115	Protein CP5		*(+)				*(-)	
**Contig3668_at**	1.00E-115	Ice recrystallization inhibition protein 1 precursor (*Triticum aestivum*)			*(-)				
Contig3861_at	_	No hit	*(-)	*(-)	*(-)		***(-)		
**Contig3863_s_at**	_	No hit		*(-)	*(-)		**(-)		
Contig398_s_at	6.00E-91	6,7-dimethyl-8-ribityllumazine synthase, chloroplast precursor						*(-)	
Contig4837_at	1.00E-52	Small nuclear ribonucleoprotein Sm D2			*(+)		**(+)		
Contig4839_at	5.00E-52	Small nuclear ribonucleoprotein Sm D2					*(-)		
**Contig6845_at**	_	No hit	*(-)				*(-)		
Contig9672_at	7.00E-52	Unknown		*(+)	*(+)		**(+)		3H (162.15)
Contig9733_at	9.00E-98	RING-H2 finger protein	*(-)						
EBem10_SQ004_D16_at	_	No hit				*(+)		*(-)	
EBpi01_SQ001_C06_at	_	No hit	***(-)				*(-)		
HB20B24r_s_at	2.00E-85	Hordoindoline b (*Hordeum vulgare*)	*(+)						
**HS04A09u_s_at**	_	No hit	***(+)				*(+)		**7H (140.21)**
HT12D12u_s_at	5.00E-48	Glutaredoxin (*Triticum aestivum*)				*(-)			
HV_CEa0001D21r2_at	_	No hit		**(+)				*(-)	2HS
HV_CEa0011J09r2_at	2.00E-18	Putative B12D protein (*Oryza sativa*)	**(-)					**(+)	1H (128.14)
HVSMEa0001B10r2_x_at	_	No hit					*(-)		
**HVSMEl0003B06r2_at**	_	No hit	**(+)				*(+)		
**HVSMEl0007F21r2_s_at**	_	No hit	**(+)						
HVSMEl0023H09r2_at	_	No hit						*(-)	
rbags14a14_at	2.00E-36	Putative prolyl endopeptidase (*Oryza sativa*)		*(-)					
rbags14a14_s_at	2.00E-36	Putative prolyl endopeptidase (*Oryza sativa*)		**(-)					6H (45.44)\

*"3d of germination"*									

Contig12150_at	_	No hit		*(-)		*(-)			7H (140.21)
**Contig12460_at**	7.00E-22	Hypothetical protein P0657H12.23 (*Oryza sativa*)		**(-)		*(-)		*(+)	
Contig13847_s_at	3.00E-85	Putative subtilisin-like serine protease (*Oryza sativa*)	**(+)					*(-)	
**Contig14046_at**	1.00E-21	Hypothetical protein OJ1756_H07 (*Oryza sativa*)			**(-)		*(-)		
Contig14709_at	3.00E-37	Os01g0579800 protein	**(-)		*(-)		*(-)		1H (138.31)
Contig159_at	3.00E-53	Histone H4	*(+)	*(+)	*(+)		***(+)		
Contig16647_at	6.00E-42	Os12g0566100 protein			**(-)		*(-)		
**Contig17218_at**	**_**	No hit	**(+)				*(+)		
Contig173_at	6.00E-54	Histone H4					*(-)		
**Contig17647_at**	2.00E-29	Os08g0100400 protein	**(-)				*(-)		
**Contig22323_at**	6.00E-38	26S protease regulatory subunit 6B	**(+)				*(+)		
**Contig2885_at**	4.00E-60	Eukaryotic translation initiation factor 5 (*Zea mays*)		*(+)		*(+)		**(-)	
**Contig3129_at**	1.00E-124	Mitochondrial prohibitin complex protein 2 (*Oryza sativa*)			**(-)		*(-)		
**Contig3668_at**	1.00E-115	Ice recrystallization inhibition protein 1 precursor (*Triticum aestivum*)			*(-)				
**Contig3863_s_at**	_	No hit		*(-)	*(-)		**(-)		
Contig4384_at	1.00E-92	DAG protein, chloroplast precursor					*(-)		5H (103.92)
Contig500_at	2.00E-53	Histone H4		*(-)	*(-)		**(-)		
Contig5185_at	1.00E-145	RNase S-like protein (*Hordeum vulgare*)	*(-)						1H (135.56)
**Contig6845_at**	_	No hit	**(-)				*(-)		
Contig7285_at	2.00E-96	Hypothetical protein P0576F08.31(*Oryza sativa*)			*(+)				6H (43.15)
Contig7399_s_at	1.00E-115	Ubiquitin carboxyl-terminal hydrolase isozyme L3		**(+)			**(+)	*(-)	6H (60.23)
HB27E22r_at	_	No hit					*(+)		
**HS04A09u_s_at**	_	No hit	***(+)				*(+)		**7H (140.21)**
**HVSMEl0003B06r2_at**	_	No hit	**(+)				*(+)		
**HVSMEl0007F21r2_s_at**	_	No hit	*(+)						

*, **, ***: Significant correlation at *p *< .05, *p *< .01, and *p *< .001, respectively. +: positive correlation, -: negative correlation. Gene annotations were exported from HarvEST:Barley version 1.68. ME: malt extract, GP: grain protein content, S/T: ratio of wort soluble protein to total malt protein, DP: diastatic power, AA: alpha-amylase activity, BG: malt beta-glucan content. Bold type indicates common genes to both time points. The genetic map locations (map loc.) were based on the barley SNP map [24].

To identify the quality-related genes whose expression became fixed through the breeding process, we first identified the differentially expressed genes between the older lines at both time points using the statistical test described above. This "older" group includes the eight lines not selected for the "most recent" group of genotypes (Dickson, M1, Cree, Bonanza, Manker, Morex, M46, and M66; Figure 1). A total of 1,094 and 416 transcripts were identified at "out of steep" and "3d of germination", respectively. Comparison of the content of these lists with the differentially expressed genes between the most recent lines (see additional file 2) allowed the removal of those entities in the overlapping region and, therefore, the identification of the genes differentially expressed only in the older members of the breeding program (1,015 at "out of steep" and 382 of the genes at "3d of germination"; Additional file 2). Examination of the correlation between gene expression and phenotypic data revealed that 693 of the 1,015 genes at "out of steep", and 206 of the 382 genes at "3d of germination", were associated with one or more of the malting quality traits (Table 6; Additional file 3). Since 86 transcripts were common in both time points, a total of 813 genes influencing malting quality were fixed during the breeding process. Most of those genes were correlated to AA (576, 70.8%), ME (545, 67%), BG (383, 47.1%), and S/T (368, 45.3%), while only 98 (12%) and 59 genes (7.3%) were associated with GP and DP, respectively (Table 6).

**Table 6:** Numbers and percentages of genes whose expression became fixed through the breeding process for six primary malting quality traits.

	ME	GP	S/T	DP	AA	BG	Total fixed
"Out of steep"	470 (67.8)	75 (10.8)	301 (43.4)	50 (7.2)	509 (73.4)	334 (48.2)	693 (100)
"3d of germination"	139 (67.5)	40 (19.4)	117 (56.8)	26 (12.6)	127 (61.7)	89 (43.2)	206 (100)
Common	64 (74.4)	17 (19.8)	50 (58.1)	17 (19.8)	60 (69.8)	40 (46.5)	86 (100)
Total	545 (67.0)	98 (12.0)	368 (45.3)	59 (7.3)	576 (70.8)	383 (47.1)	813 (100)

Percentages are shown in parentheses. Common genes to both time points, as well as total number of correlated genes considering both time points are also shown. Note that a given transcript may correlate with multiple malt quality traits. ME: malt extract, GP: grain protein content, S/T: ratio of wort soluble protein to total malt protein, DP: diastatic power, AA: alpha-amylase activity, BG: malt beta-glucan content.

## Discussion

The University of Minnesota six-rowed malting barley breeding program has successfully used advanced cycle breeding for decades, releasing important malting cultivars. However, this breeding method has led to a reduction in genetic diversity over time that has mostly occurred in genomic regions associated with traits under selection, including disease-resistance genes and favorable malting quality characteristics [3]. Since these reductions will predictably limit future breeding progress, it is necessary to assess the existing diversity in the germplasm pool available for future improvements in traits of interest. Although microarray technologies have been previously used to characterize the transcript profiling in different barley cultivars [17,19,22], they have not been used to evaluate malting lines from a breeding program. The selection of 15 lines representing the University of Minnesota malting barley breeding program and their transcriptome analysis at two stages of germination has provided the ability to examine gene expression diversity within the program, to identify the amount of that gene expression diversity still available for further improvement, and to find genes potentially associated with malting quality.

### Transcriptome variation among the breeding program is correlated with genetic distance

Transcriptome comparison of the 15 lines identified a total of 1,442 differentially expressed transcripts at time point "out of steep". A lower number of genes (851) was found to be differentially expressed between the lines at "3d of germination", indicating that, as the germination process progresses, gene expression differences between malting genotypes become smaller. A reduced amount of differentially expressed genes was also found after 3 days of germination when two malting genotypes were compared in a previous study [19]. Pair-wise comparisons revealed that the number of differentially expressed genes between any two genotypes across time points varied from 3 to 1,005 (Tables 1 and 2). Dickson and Bonanza were the lines with the largest number of differentially expressed genes, an expected result since they were two parent lines of this study that were introduced to the Minnesota breeding program from North Dakota State University and Agriculture & Agri-Food Canada [3]. The variation found in the transcriptome of these 15 lines is smaller than the transcript-level variation found within seven *Arabidopsis thaliana *accessions [15], but is within the range of transcript-level variation found between 5 maize inbreds [16] and within 4 malting barley varieties [17]. This is probably due to the more closely related genotypes used in the last two studies. However, further studies evaluating gene expression diversity in barley would be required to assess whether values presented herein are representative of the particular species or are due to the closely related genotypes used in the study.

The relationship of genetic to gene expression differences between genotypes have also been examined in Arabidopsis [15] and maize [16] to determine if DNA sequence polymorphism are associated with gene expression diversity. In this study, pair-wise genetic similarity indexes were calculated between the 15 lines after their genotyping with 1,524 SNP markers (Table 3). Their high values, ranging from 0.829 to 0.994, showed the narrow genetic base of the University of Minnesota breeding program. In accordance with the Kliebenstein et al. [15] and Stupar et al. [16] studies, we found a significant correlation between gene expression and genetic diversity (Figure 2).

### Gene expression diversity is still present after forty years of advanced cycle breeding

Several studies have analyzed the decrease in genetic diversity due to breeding in North American six-rowed barley cultivars [3,5,7,13,14,29]. However, the repercussions of consecutive cycles of breeding on gene expression diversity were not examined. Identification of the differentially expressed genes between each line and Lacey and their placement on the figure that represents the pedigree of the lines (Figure 1), allowed us to visualize how advanced cycle breeding has affected gene expression diversity. Gene expression diversity is reduced through the breeding process, as shown by the decreased numbers of differentially expressed genes between the last cultivar released by the breeding program and its closer relatives. Genetic diversity is also reduced as the breeding program progresses, as can be seen by the increase in the GS indexes (Figure 1). However, Robust had an unexpectedly lower GS index that was not correlated with its gene expression data. Two previous studies, based on information from SSR molecular markers [3] and coefficient of parentages [5], clustered Robust together with Lacey, which suggests that our gene expression data are probably a better measure of the relationship between Robust and Lacey. Although both genetic and gene expression values of M66 were also unexpected, they support the previous study of Condón et al. [3], which placed M66 out of the contemporary group of elite lines. Interestingly, our data showed that the level of gene expression diversity between one of the parental lines (Dickson) and Lacey is much higher than between the other parental line (Bonanza) and Lacey, suggesting that Bonanza made a higher contribution to Lacey than Dickson. Although variation in gene expression was reduced in this breeding program, the gene expression patterns have not been fixed between the lines released in the last decade. These results indicate that four decades of breeding have not eliminated the gene expression differences and imply the possibility of obtaining further improvements from crossing elite lines from the breeding program.

### Identification of target genes for enhancing malting quality

Improvement of the complex phenotype "malting quality" is the main goal of the University of Minnesota breeding program and, therefore, most of the reduction in gene expression diversity will be predictably associated with this phenotype. The high number of quality-related genes (813) whose expression has been fixed during the breeding progress (Table 6; Additional file 3) supports this assumption. Malt extract (ME) is probably the single most important parameter for maltsters and brewers due to its influence on ethanol production and hence the quantity of beer produced. A previous study including the genotypes used in this work detected significant gains in this trait during the advanced cycle breeding [13]. Our phenotypic data, showing a ME increase of around 3% (Table 4), together with the finding of a high amount (67%) of ME-correlated genes with a fixed expression in the most recent lines of the program (Table 6), corroborates the selection pressure that has been exerted on this trait. In agreement with this previous work, an increase in the AA levels over time was also detected in our data obtained from finished malts derived from the microarray experiment (Table 4). This trait also has a similarly high number (70.8%) of associated genes with fixed transcript levels through the breeding process (Table 6). Unlike ME, selecting for higher AA was indirect and maybe due to the selection for related traits such as dormancy [13,30]. Although many S/T-correlated genes exhibited fixed expression during the breeding process (Table 6), little improvement was observed for this trait (Table 4). Both high grain protein content (GP) and high wort β-glucan (BG) concentrations cause problems during brewing, and reductions in both parameters are desirable in malting barley varieties. Significant decreases in both traits have been reported during advanced cycle breeding [13]. An overall reduction in BG was also observed in our data (Table 4) and a high number of BG-correlated genes with a fixed expression were detected (Table 6; Additional file 3). However, no reduction over time was observed in our GP values (Table 4). The low correlation (0.30) between malting quality data derived from grain used in the the microarray experiment and previous field data for GP ([13]; Additional file 1) is likely due to the limited variation for this trait among the lines in the microarray experiment.

To identify the gene expression diversity still available for future enhancements on quality traits within the program, we focused on the genes differentially expressed between the most recent lines that were correlated to any of the six quality-assurance parameters. Considering both time points, 49 genes constituting potential targets for the improvement of malting quality were identified (Table 5). From the quality traits evaluated, the greatest potential for targeted improvements can be expected for AA and ME since they had the highest number of associated genes. Interestingly, these two traits (AA and ME) for which the greatest increases have been achieved in this advanced cycle breeding program, and for which the most genes have fixed their expression during the breeding program, are also the ones associated with the greatest number of specific target genes for future improvements. This is in agreement with a previous study suggesting that there are unknown genes for ME segregating in the breeding population, since the known chromosomal regions associated with ME had been fixed in these elite lines while the phenotypic variance had not been reduced over time [13]. These authors also suggested that new genetic diversity for AA must have been introduced to the program to explain its variable pattern of phenotypic variance over the four decades of breeding. In the case of GP, BG and S/T, gene expression diversity analysis has also suggested targets to be exploited for future gains, as shown by the relatively high number of correlated candidate genes (Table 5). However, the smallest improvements by intercrossing these elite lines can be expected for DP, given the low number (6) of candidate genes (Table 5). Moreover, a few DP-associated genes (59) fixed their expression during the breeding process (Table 6), indicating a low diversity for this trait within the breeding program. Although DP values exceed current AMBA criteria, a future improvement of this trait would require additional diversity introduced into the University of Minnesota gene pool.

Focusing on the 49 candidate transcripts, none of them corresponded to the well-characterized genes controlling malting quality in barley such as α- and β-amylases, α-glucosidase, carboxypeptidases and cysteine endoproteinases [31,32]. This was an expected finding since the expression of those genes would have been fixed very quickly in the elite lines, as observed by the numerous genes coding for these proteins that were found to be fixed through the breeding process (Table 6; Additional file 3). Similarly, none of our candidate genes was coincident with the genes correlated with malting quality traits in recent studies [17,22]. However, the barley aleurone transcript B12D identified at "out of steep" (Table 5) was also identified by [21] as an abundant transcript expressed during malting. This gene, which is known to be expressed during germination and to have GA response elements in its promoter [33], is negatively correlated to ME and positively to BG. Since desirable quality characteristics include high ME and low BG values [34], the expression of this gene in our lines has a negative influence on these two malting quality traits.

Historically, the greatest emphasis has been placed on the role of cysteine-class proteinases for overall endopeptidase activity [34] and their ability to degrade Hordein, the proline-rich storage protein, with more recent studies suggesting a role for metalloproteinases in protein solubilization [35]. However, some endopeptidases belonging to the serine family have been identified during germination and malting [19,36]. Additional effects of the serine endopeptidases on malting quality have been suggested due to their ability to degrade malt beta-amylase, which would have an indirect effect in reducing DP [37,38]. Interestingly, three of our candidate genes encoded serine-type endopeptidases (Table 5). One of them (subtilisin-like serine protease) was positively correlated to ME and negatively correlated to BG. This highlights the complexity of the roles of the various proteinases in affecting malting quality since Schmitt et al. [37] found overall negative correlations between bulk serine endoproteinase activity and ME along with a positive correlation between bulk serine proteinase activity and BG. The other two serine class proteinases (prolyl endopeptidases) showed negative correlations with GP. It is attractive to speculate that the negative link between the prolyl endopeptidases and GP is through the ability of the prolyl peptidases to degrade the proline-rich storage proteins (Hordeins) in barley. There has been significant interest recently in degradation of prolamins from a number of cereals, including barley, by prolyl endopeptidases both due to the involvement of the prolamins in haze formation in beer (see [39]), as well as involvement in dietary sensitivity (Celiac sprue) to proline-containing peptides [40-42]. While prolyl endopeptidases have not previously been shown to function in malting, the results here may suggest that they could have a role in malting quality by affecting grain protein levels during grain maturation.

Additionally, barley hordoindoline b was positively correlated with ME at "out of steep" (Table 5). These proteins have been found to be associated with grain hardness [43], which is significantly correlated with ME [44]. While many of the postulated roles of these genes in affecting malting quality are still speculative, in the absence of a demonstrated functional path from the gene product to the final malting quality phenotype, our results provide a number of novel hypotheses for selection targets for further improvements in malting quality.

## Conclusions

In this study, we analyzed the transcriptome of barley genotypes belonging to a historically important breeding program. Our results indicate that the use of advanced cycle breeding in the University of Minnesota six-rowed malting barley breeding program has had an effect on gene expression diversity, with an overall reduction in the number of differentially expressed genes over time. However, gene expression diversity is still present after forty years of advanced cycle breeding and further improvements in malting quality traits can still be obtained from intercrossing these elite lines, as shown by the 49 quality-associated genes exhibiting differential expression between them. However, management of this existing diversity will be required for long-term breeding progress, and future introgressions of exotic or wild germplasm may be required for managing new problems affecting this crop. Availability of new tools such as high-throughput molecular marker technology will help breeders to actively manage the levels of diversity in their programs while at the same time make progress for important traits.

## Authors' contributions

MMA analyzed and interpreted the data, and wrote the paper; YX, AB, SC and HB obtained the data; GJM, KPS and MRS designed the experiment, interpreted the data and edited the paper. All the authors read and approved the final manuscript.

## Supplementary Material

Additional file 1 **Prior malting quality data for the fifteen barley genotypes**. Data were collected from five field trials conducted in Minnesota in 2002 and 2003 (data previously published in [13]). Click here for file

Additional file 2 **Venn diagrams of the differentially expressed genes between the most recent and older genotypes at both time points**. The figure shows the intersection among three data sets corresponding to: all probe sets on the Barley1 GeneChip, the differentially expressed genes between the "most recent" lines, and the genes differentially expressed between the "older" lines. Click here for file

Additional file 3 **Quality-related genes whose expression was fixed through the breeding process**. Differentially expressed genes only between the "older" lines that are correlated with any of the six malting quality traits. Significant correlations for each of the six quality-assurance parameters are shown (*: *p *< 0.05, **: *p *< 0.01 and ***: *p *< 0.001), together with the sign of the correlation (+: positive correlation, -: negative correlation). Bold type indicates common genes to both time points. Click here for file

## References

[B1] Bernardo R Breeding for quantitative traits in plants 2002 Woodbury, MN: Stemma Press

[B2] Yu J Bernardo R Changes in genetic variance during advanced cycle breeding in maize Crop Sci 2004 44 405 410 10.2135/cropsci2004.1502

[B3] Condón F Gustus C Rasmusson DC Smith KP Effect of advanced cycle breeding on genetic diversity in barley breeding germplasm Crop Sci 2008 48 1027 1036 10.2135/cropsci2007.07.0415

[B4] Martin JM Blake TK Hockett EA Diversity among North American spring barley cultivars based on coefficients of parentage Crop Sci 1991 31 1131 1137 10.2135/cropsci1991.0011183X003100050009x

[B5] Mikel MA Kolb FL Genetic diversity of contemporary North American Barley Crop Sci 2008 48 1399 1407 10.2135/cropsci2008.01.0029

[B6] National Research Council Agricultural crop issues and policies 1993 Washington, DC: National Academy Press

[B7] Rasmusson DC Phillips RL Plant breeding progress and genetic diversity from de novo variation and elevated epistasis Crop Sci 1997 37 303 310 10.2135/cropsci1997.0011183X003700020001x

[B8] Fu YB Somers DJ Genome-wide reduction of genetic diversity in wheat breeding Crop Sci 2009 49 161 168 10.2135/cropsci2008.03.0125

[B9] Burger WC LaBerge DE Rasmusson DC Malting and Brewing Quality Barley, Agronomy Monograph. No. 26 1985 Madison, WI: ASA-CSSA-SSSA 367 401

[B10] Fox GP Panozzo JF Li CD Lance RCM Inkerman PA Henry RJ Molecular basis of barley quality Aust J Agric Res 2003 54 1081 1101 10.1071/AR02237

[B11] Hayes PM Jones BL Logue S Malting quality from a QTL perspective8th International Barley Genetics Symposium, 22-27 October 2000 2000 Adelaide, South Australia 99 105

[B12] Szűcs P Blake VC Bhat PR Chao S Close TJ Cuesta-Marcos A Muehlbauer GJ Ramsay L Waugh R Hayes PM An integrated resource for barley linkage map and malting quality QTL alignment Plant Genome 2009 2 1 7 10.3835/plantgenome2009.02.0001ed

[B13] Condón F Rasmusson DC Schiefelbein E Velasquez G Smith KP Effect of advanced cycle breeding on genetic gain and phenotypic diversity in barley breeding germplasm Crop Sci 2009 49 1751 1761 10.2135/cropsci2008.10.0585

[B14] Horsley RD Schwarz PB Hammond JJ Genetic diversity in malt quality of North American six-rowed spring barley Crop Sci 1995 35 113 118 10.2135/cropsci1995.0011183X003500010021x

[B15] Kliebenstein DJ West MAL van Leeuwen H Kim K Doerge RW Michelmore RW St Clair DA Genomic survey of gene expression diversity in *Arabidopsis thaliana* Genetics 2006 172 1179 1189 10.1534/genetics.105.049353 16204207 PMC1456216

[B16] Stupar RM Gardiner JM Oldre AG Haun WJ Chandler VL Springer NM Gene expression analyses in maize inbreds and hybrids with varying levels of heterosis BMC Plant Biol 2008 8 33 10.1186/1471-2229-8-33 18402703 PMC2365949

[B17] Lapitan NLV Hess A Cooper B Botha AM Badillo D Iyer H Menert J Close TJ Wright L Hanning G Tahir M Lawrence C Differentially expressed genes during malting and correlation with malting quality phenotypes in barley (*Hordeum vulgare *L.) Theor Appl Genet 2009 118 937 952 10.1007/s00122-008-0951-8 19132335

[B18] Potokina E Sreenivasulu N Altschmied L Michalek W Graner A Differential gene expression during seed germination in barley (*Hordeum vulgare *L.) Funct Integr Genomics 2002 2 28 39 10.1007/s10142-002-0050-x 12021848

[B19] Muñoz-Amatriaín M Cistué L Xiong Y Bilgic H Budde AD Schmitt MR Smith KP Hayes PM Muehlbauer GJ Structural and functional characterization of a winter malting barley Theor Appl Genet 2010 120 971 984 10.1007/s00122-009-1225-9 19960335

[B20] Watson L Henry RJ Microarray analysis of gene expression in germinating barley embryos (*Hordeum vulgare *L.) Funct Integr Genomics 2005 5 155 162 10.1007/s10142-005-0133-6 15714320

[B21] White J Pacey-Miller T Crawford A Cordeiro G Barbary D Bundock P Henry R Abundant transcripts of malting barley identified by serial analysis of gene expression (SAGE) Plant Biotech J 2006 4 289 301 10.1111/j.1467-7652.2006.00181.x 17147635

[B22] Potokina E Caspers M Prasad M Kota R Zhang H Sreenivasulu N Wang M Graner A Functional association between malting quality trait components and cDNA array based expression patterns in barley (*Hordeum vulgare *L.) Mol Breeding 2004 14 153 170 10.1023/B:MOLB.0000038004.21342.3c

[B23] Benjamini Y Hochberg Y Controlling the false discovery rate: A practical and powerful approach to multiple testing J R Stat Soc Ser B 1995 57 289 300

[B24] Close TJ Bhat PR Lonardi S Wu Y Rostoks N Ramsay L Druka A Stein N Svensson JT Wanamaker S Bozdag S Roose ML Moscou MJ Chao S Varshney R Szőcs P Sato K Hayes PM Matthews DE Kleinhofs A Muehlbauer GJ DeYoung J Marshall DF Madishetty K Fenton RD Condamine P Graner A Waugh R Development and implementation of high-throughput SNP genotyping in barley BMC Genomics 2009 10 582 10.1186/1471-2164-10-582 19961604 PMC2797026

[B25] Nei M Li W Mathematical model for studying genetic variation in terms of restriction endonucleases Proc Natl Acad Sci USA 1979 76 5269 5273 10.1073/pnas.76.10.5269 291943 PMC413122

[B26] Rostoks N Borevitz JO Hedley PE Russell J Mudie S Morris J Cardle L Marshall DF Waugh R Single-feature polymorphism discovery in the barley transcriptome Genome Biol 2005 6 R54 10.1186/gb-2005-6-6-r54 15960806 PMC1175974

[B27] Xu WW Cho S Yang SS Bolon YT Bilgic H Jia H Wiong Y Muehlbauer GJ Single-feature polymorphism discovery by computing probe affinity shape powers BMC Genetics 2009 10 48 10.1186/1471-2156-10-48 19709416 PMC2746803

[B28] Close TJ Wanamaker S Caldo RA Turner SM Ashlock DA Dickerson JA Wing RA Muehlbauer GJ Kleinhofs A Wise RP A new resource for cereal genomics: 22 K barley GeneChip comes of age Plant Physiol 2004 134 960 968 10.1104/pp.103.034462 15020760 PMC389919

[B29] Schwarz PB Horsley RD Malt quality improvement in North American six-rowed barley cultivars since 1910 J Am Soc Brew Chem 1995 53 14 18

[B30] Li CD Tarr A Lance RC Harasymow S Uhlmann J Westcot S Young KJ Grime CR Cakir M Broughton S Appels R A major QTL controlling seed dormancy and pre-harvest sprouting/grain α-amylase in two-rowed barley (*Hordeum vulgare *L.) Aust J Agric Res 2003 54 1303 1313 10.1071/AR02210

[B31] Hayes MP Castro A Marquez-Cedillo L Corey A Henson C Jones BL Kling J Mather D Matus I Rossi C Sato K von Bothmer R, van Hintum T, Knüpffer H Genetic diversity for quantitatively inherited agronomic and malting quality traits Diversity in Barley (Hordeum vulgare) 2003 Sato. Amsterdam, The Netherlands: Elsevier Science 201 226 full_text

[B32] Potokina E Prasad M Malysheva L Röder MS Graner A Expression genetics and haplotype analysis reveal *cis *regulation of serine carboxypeptidase I (*Cxp1*), a candidate gene for malting quality in barley (*Hordeum vulgare *L.) Funct Integr Genomics 2006 6 25 35 10.1007/s10142-005-0008-x 16283224

[B33] Steinum TM Berner HS Stacy RAP Salehian Z Aalen RB Differential regulation of the barley (*Hordeum vulgare*) transcripts B22E and B12D in mature aleurone layers Physiol Plantarum 1998 102 337 345 10.1034/j.1399-3054.1998.1020301.x

[B34] Bamforth CW Barclay AHP McGregor A, Bhatty RS Malting technology and the uses of malt Barley: chemistry and technology 1993 St. Paul: American Association of cereal Chemists 297 354

[B35] Jones BL Budde AD How various malt endoproteinase classes affect wort roluble protein levels J Cereal Sci 2005 41 95 106 10.1016/j.jcs.2004.09.007

[B36] Fontanini D Jones BL SEP1 - a subtilisin-like serine endopeptidase from germinated seeds of *Hordeum vulgare *L. cv. Morex Planta 2002 215 885 893 10.1007/s00425-002-0823-4 12355148

[B37] Schmitt MR Budde AD Improved methods for high-throughput extraction and assay of green barley malt proteinase activity facilitating examination of proteinase activity across large-scale populations Cereal Chem 2007 84 313 319 10.1094/CCHEM-84-4-0313

[B38] Schmitt MR Marinac L *Beta*-amylase degradation by serine endoproteinases from green barley malt J Cereal Sci 2008 47 480 488 10.1016/j.jcs.2007.06.002

[B39] Bamforth CW Current perspectives on the role of enzymes in brewing J Cereal Sci 2009 50 353 357 10.1016/j.jcs.2009.03.001

[B40] Ehren J Govindarajan S Morón B Minshull J Khosla C Protein engineering of improved prolyl endopeptidases for celiac sprue therapy Protein Eng Des Sel 2008 21 699 707 10.1093/protein/gzn050 18836204 PMC2583057

[B41] Mitea C Havenaar R Drijfhout JW Edens L Dekking L Koning F Efficient degradation of gluten by a prolyl endoprotease in a gastrointestinal model: implications for celiac disease Gut 2008 57 25 32 10.1136/gut.2006.111609 17494108

[B42] Stepniak D Spaenih-Dekking L Mitea C Moester M de Ru A Baak-Pablo R van Veelen P Edens L Koning F Highly efficient gluten degradation with a newly identified prolyl endopeptidase: implications for celiac disease Am J Physiol Gastrointest Liver Physiol 2006 291 G621 G629 10.1152/ajpgi.00034.2006 16690904

[B43] Beecher B Bowman J Martin JM Bettge AD Morris CF Blake TK Giroux MJ Hordoindolines are associated with a major endosperm-texture QTL in barley (*Hordeum vulgare*) Genome 2002 45 584 591 10.1139/g02-008 12033628

[B44] Nagamine T Sekiwa T Yamaguchi E Oozeki M Kato T Relationship between quality parameters and SKCS hardness index in malting barley J Inst Brew 2009 115 292 295

