# Supplementary material for: Transcriptome analysis of a barley breeding program examines gene expression diversity and reveals target genes for malting quality improvement
Source: BMC Genomics. 2010 Nov 23;11:653. doi: 10.1186/1471-2164-11-653 (PMC3091773; doi:10.1186/1471-2164-11-653)
Supplement: Additional file 2 — Venn diagrams of the differentially expressed genes between the most recent and older genotypes at both time points. The figure shows the intersection among three data sets corresponding to: all probe sets on the Barley1 GeneChip, the differentially expressed genes between the "most recent" lines, and the genes differentially expressed between the "older" lines. [file 1471-2164-11-653-S2.PPT]

## Slide 1
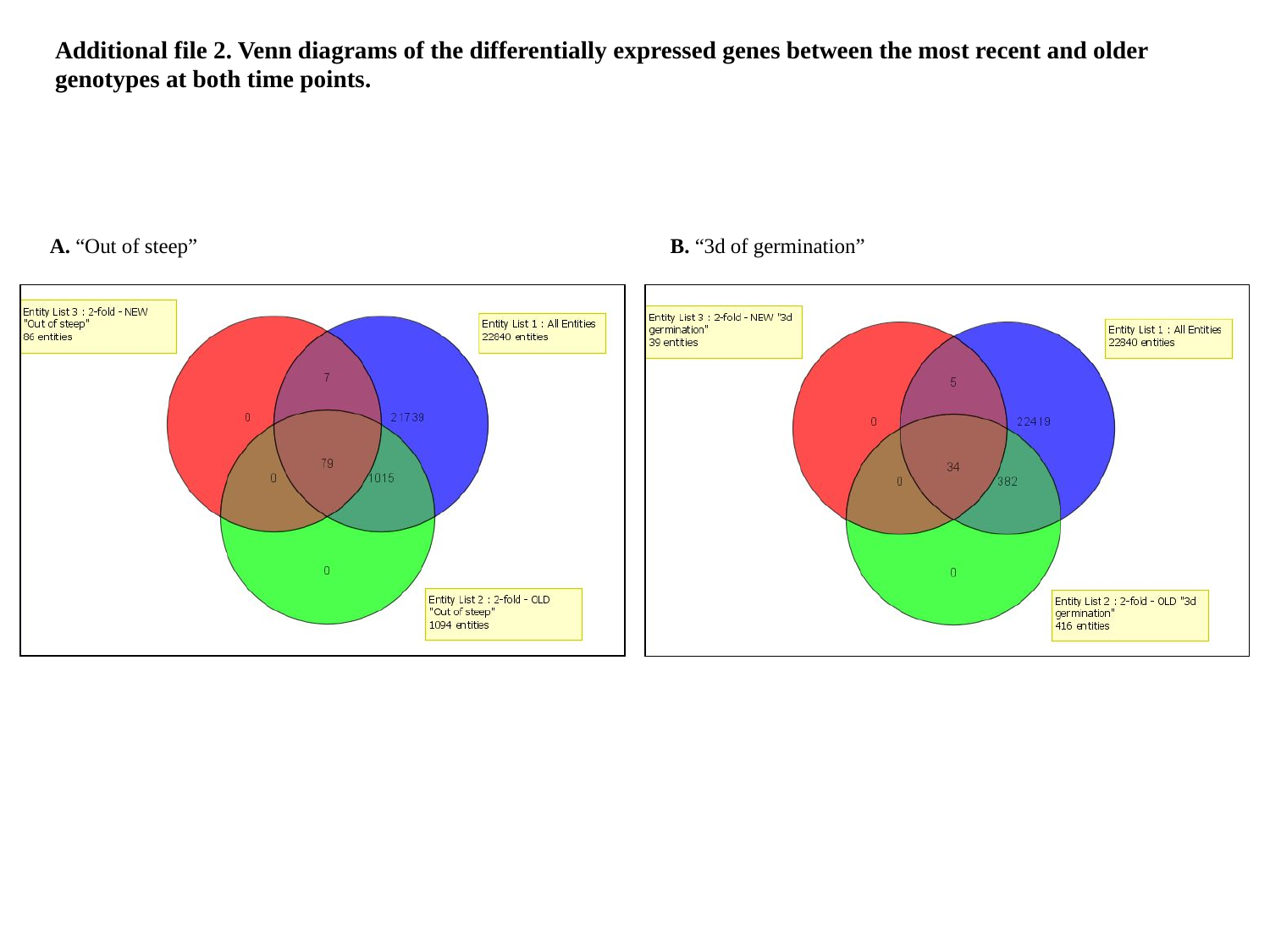

Additional file 2. Venn diagrams of the differentially expressed genes between the most recent and older genotypes at both time points.
A. “Out of steep”
B. “3d of germination”
